# The relationship between childhood emotional neglect and emotional eating: the chain mediating role of alexithymia and difficulties in emotion regulation

**DOI:** 10.3389/fpsyt.2026.1895255

**Published:** 2026-08-19

**Authors:** Qin Guan, Xiaowei Liu, Chunlu Li, Yongying Yang, Cui Lv

**Affiliations:** 1 College of Medical Humanities, Guizhou Medical University, Guiyang, China; 2 College of Basic Medical Sciences, Guizhou Medical University, Guiyang, China; 3 Department of Orthopedic and Sports Medicine, Jinan People’s Hospital, Jinan, China; 4 Department of Psychiatry and Psychology, Affiliated Hospital of Guizhou Medical University, Guiyang, China

**Keywords:** alexithymia, chain mediation, childhood emotional neglect, difficulties in emotion regulation, emotional eating

## Abstract

**Background:**

Emotional eating is prevalent across various weight groups and has a significant impact on individuals’ emotion regulation and healthy eating behaviors. As an early adverse experience, childhood emotional neglect may be associated with greater emotional eating tendencies, potentially through poorer emotion recognition and emotion regulation; however, the underlying pathways remain unclear.

**Objective:**

To examine the relationship between childhood emotional neglect and emotional eating among college students, and to test the independent and chain mediating effects of alexithymia and difficulties in emotion regulation.

**Methods:**

A cross-sectional survey was conducted with 861 college students. Participants completed the Emotional Neglect subscale of the Childhood Trauma Questionnaire (CTQ), the Emotional Eating Scale (EES), the Toronto Alexithymia Scale (TAS), and the Difficulties in Emotion Regulation Scale (DERS). Correlation analysis, regression analysis, and the Bootstrap method were used to examine mediating effects.

**Results:**

Childhood emotional neglect was significantly positively correlated with alexithymia (*r* = 0.242, *P* < 0.01) and difficulties in emotion regulation (*r* = 0.233, *P* < 0.01), but not with emotional eating (*r* = 0.011, *P* > 0.05). Emotional eating was significantly positively correlated with alexithymia (*r* = 0.160, *P* < 0.01) and difficulties in emotion regulation (*r* = 0.169, *P* < 0.01). Alexithymia was significantly positively correlated with difficulties in emotion regulation (*r* = 0.609, *P* < 0.01). Mediation analysis revealed a significant total indirect effect (effect = 0.1994, 95% CI [0.1118, 0.2985]). The independent mediating effect of alexithymia (0.0906, 95% CI [0.0034, 0.1832]), the independent mediating effect of difficulties in emotion regulation (0.0419, 95% CI [0.0060, 0.0895]), and the chain mediating effect of both (0.0668, 95% CI [0.0124, 0.1306]) were all significant. Neither the direct effect nor the total effect was significant.

**Conclusions:**

Childhood emotional neglect was not directly associated with emotional eating among college students, but was indirectly associated with emotional eating through alexithymia, difficulties in emotion regulation, as well as their sequential indirect pathway.

## 1 Introduction

Emotional eating refers to eating behavior that occurs in the absence of physiological hunger, driven by the need to alleviate negative emotions such as anxiety and depression (1). Essentially, it is a coping strategy that uses food to regulate emotions. Existing research has shown that emotional eating is not only common among overweight and obese populations, where it has been associated with higher body weight and greater metabolic risk over time (2, 3), but is also prevalent among individuals with normal weight (4). For college students, although emotional eating may not yet manifest in obvious metabolic consequences, it may reflect difficulties in emotional coping and health behavior management, and may be associated with grater vulnerability to unhealthy dietary patterns and weight-related problems.

From a developmental perspective, college students are in a critical transitional period from late adolescence to early adulthood, facing multiple developmental tasks in areas such as identity exploration, autonomy development, and interpersonal adaptation (5). Meanwhile, their lifestyles and behavioral habits are gradually shifting from external regulation to self-management (6), however, their emotion regulation and behavioral control capacities are not yet fully mature. Accordingly, when confronted with stressors such as academic competition and interpersonal difficulties, they may be more likely to adopt short-term coping strategies that provide immediate gratification (7). According to stress-coping theory, emotional eating can be viewed as a short-term relief strategy employed by individuals under pressure and in negative emotional situations (8). Therefore, investigating the factors and potential processes associated with emotional eating in non-clinical college student populations is of great significance for understanding their emotional and behavioral adaptation issues, as well as for identifying early health risks.

In recent years, researchers have increasingly focused on early adverse experiences as potential risk factors for emotional eating, with childhood trauma identified as a particularly important antecedent (9). Childhood trauma encompasses five subtypes: physical abuse, physical neglect, emotional abuse, emotional neglect, and sexual abuse (10), each of which may be associated with subsequent psychological adaptation and behavioral development. Among these subtypes, childhood emotional neglect warrants particular attention. Emotional neglect refers to a chronic failure by caregivers to respond to a child’s emotional needs, which may limit opportunities for emotional socialization, including the recognition, expression, and regulation of emotions, and may be associated with a greater tendency toward maladaptive emotional coping strategies (11). According to emotion socialization theory, the development of children’s emotional competence does not occur naturally; rather, it depends on the responses, mirroring, and supportive guidance provided by primary caregivers regarding their emotional experiences (12). If children are chronically exposed to environments where their emotional needs remain unmet, they may show poorer abilities in identifying, expressing, and regulating emotions. Previous studies have linked childhood emotional neglect to a range of maladaptive outcomes, including depression, anxiety, low self-esteem, insecure attachment, and impulsive behavior (13–15). Given that emotional eating is typically conceptualized as an emotion-related behavioral coping strategy, childhood emotional neglect may be relevant to individual differences in emotional eating.

However, whether a stable direct relationship exists between childhood emotional neglect and emotional eating remains inconclusive. One study found that after controlling for war exposure and PTSD symptoms, the association between emotional neglect and emotional eating was no longer significant, with effects operating primarily through emotional dysregulation (9). Similarly, another study demonstrated that when other adverse childhood experiences were controlled, emotional neglect was not significantly associated with binge eating (16). Although binge eating and emotional eating are not entirely equivalent, both findings suggest that the association between childhood emotional neglect and eating problems may not present as a stable direct relationship, but may instead be related to specific emotion-related processes. Therefore, further investigation of the potential mediating roles of emotion-related factors in the association between childhood emotional neglect and emotional eating is warranted.

Alexithymia may represent one important factor that helps explain the association between childhood emotional neglect and emotional eating. Alexithymia is characterized by difficulties in identifying, describing, and understanding emotions (17). Children raised in emotionally neglectful environments, characterized by limited emotional responsiveness and feedback, may show poorer emotional responsiveness and feedback, may show poorer emotion recognition and representation abilities, which may in turn be associated with higher levels of alexithymia (18). Furthermore, individuals with alexithymia often struggle to differentiate emotional arousal from physiological states and may be more likely to rely on external, immediate strategies—such as eating—to cope with internal discomfort (19). Based on this theoretical rationale, we propose Hypothesis 1: Alexithymia may mediate the association between childhood emotional neglect and emotional eating.

In addition to alexithymia, difficulties in emotion regulation may constitute another important factor associated with the link between childhood emotional neglect and emotional eating. Difficulties in emotion regulation refer to deficits in the awareness, acceptance, and management of emotions, as well as inflexibility in employing regulatory strategies (20, 21). Previous research has indicated that inadequate early emotional environments may be associated with poorer subsequent emotion regulation functioning, and that emotion regulation difficulties are associated with various maladaptive behaviors. Under conditions of negative affect, individuals may be more likely to adopt avoidance, suppression, or eating behaviors as temporary means of relief (20). Compared with childhood emotional neglect, difficulties in emotion regulation may be more closely related to current emotional eating at the level of concurrent emotional functioning. Accordingly, we propose Hypothesis 2: Difficulties in emotion regulation may mediate the association between childhood emotional neglect and emotional eating.

Furthermore, alexithymia and difficulties in emotion regulation may not function independently, but may instead show a theoretically derived sequential association. According to Gross’s process model of emotion regulation, effective emotion regulation begins with accurate identification of emotions (22). If individuals have difficulty recognizing and understanding their emotions, they may also have difficulty selecting appropriate regulatory strategies and may report greater emotion regulation difficulties. Previous research has documented a significant positive association between alexithymia and difficulties in emotion regulation (23), yet the two are not the same psychological construct. Alexithymia primarily reflects deficits in an individual’s ability to recognize, differentiate, and represent emotions, representing an impairment in the early stage of emotional processing (16); whereas difficulties in emotion regulation mainly manifest as functional impairments in the subsequent stages after emotions have been activated (20). In other words, the former emphasizes whether one is aware of what emotion one is experiencing at the moment, while the latter emphasizes whether one can effectively manage emotions after recognizing them. This theoretical distinction suggests that the two represent different levels of impaired emotional functioning. Thus, within the hypothesized association between childhood emotional neglect and emotional eating, alexithymia may be more closely related to earlier stages of emotional processing and may be linked to emotional eating through its association with difficulties in emotion regulation. Based on this reasoning, we propose **Hypothesis 3**: Alexithymia and difficulties in emotion regulation may sequentially mediate the association between childhood emotional neglect and emotional eating.

In summary, childhood emotional neglect, as an adverse developmental experience, may not be directly associated with emotional eating, but may instead be indirectly related to emotional eating through emotion-processing and emotion-regulation-related factors. Specifically, alexithymia reflects difficulties in emotion identification, comprehension, and expression, and may be one factor associated with the link between childhood emotional neglect and current maladaptive eating behavior. Difficulties in emotion regulation may be more closely associated with current emotional eating, given their relevance to responding to negative emotional states. These two factors may also show a theoretically proposed sequential association. Based on this theoretical framework, the present study aims to test a chain mediation model examining the roles of alexithymia and difficulties in emotion regulation in the association between childhood emotional neglect and emotional eating, thereby contributing to the understanding of factors associated with emotional eating and providing preliminary implications for relevant intervention efforts.

## 2 Participants and methods

### 2.1 Participants

This study employed a cross-sectional design. From March to April 2026, a cluster sampling method was used to recruit undergraduate students from a university in Guizhou Province, China. Questionnaires were administered in person across different classes on campus. A total of 1,000 questionnaires were distributed, of which 861 were valid, yielding an effective response rate of 86.10%.

The sample comprised 288 men (33.4%) and 573 women (66.6%). Regarding academic year, there were 338 first-year students (39.3%), 250 second-year students (29.0%), 171 third-year students (19.9%), and 102 fourth-year students (11.8%). In terms of family residence, 569 participants (66.1%) were from rural areas and 292 (33.9%) from urban areas. With respect to only-child status, 98 participants (11.4%) were only children and 763 (88.6%) had siblings. Regarding family structure, 740 participants (85.9%) were from intact families, 70 (8.1%) from divorced families, 39 (4.5%) from restructured families, and 12 (1.4%) from other family structures. Based on body mass index (BMI), 190 participants (22.1%) were underweight, 556 (64.6%) were within the normal range, 94 (10.9%) were overweight, and 21 (2.4%) were obese.

### 2.2 Measures

#### 2.2.1 Demographic questionnaire

A self-designed demographic questionnaire was used to collect participants’ basic information, including gender, academic year, family residence, only-child status, family structure, and BMI.

#### 2.2.2 Childhood emotional neglect subscale

The Childhood Emotional Neglect (CEN) subscale of the Childhood Trauma Questionnaire (CTQ), revised in Chinese by Zhao and colleagues (24), was used to assess the severity of childhood emotional neglect. This subscale consists of 5 items rated on a 5-point Likert scale. All items were reverse-scored, with higher scores indicating more severe childhood emotional neglect. In the present study, the Cronbach’s α coefficient for this subscale was 0.936.

#### 2.2.3 Emotional eating scale

The Chinese version of the Emotional Eating Scale (EES-C), revised by Zhu and colleagues (25), was used to assess the severity of emotional eating. The scale comprises four dimensions: depression, anxiety, hostility, and positive emotion, designed to evaluate eating responses to depressive, hostile, anxious, and positive emotional states. The EES-C contains 23 items rated on a 5-point Likert scale (1 = not at all, 5 = very strong). The total score was computed by summing all item scores, with higher scores indicating greater frequency of emotional eating. In the present study, the Cronbach’s α coefficient for the total scale was 0.936.

#### 2.2.4 Toronto alexithymia scale

The Chinese version of the Toronto Alexithymia Scale (TAS-20), revised by Yuan and colleagues (26), was used to assess alexithymia. This is the most widely used instrument for assessing alexithymia in China and comprises three dimensions: difficulty identifying feelings, difficulty describing feelings, and externally oriented thinking. The scale contains 20 items rated on a 5-point Likert scale (1 = strongly disagree, 5 = strongly agree). Items 4, 5, 10, 18, and 19 were reverse-scored. The total score ranges from 20 to 100, with scores ≤ 50 indicating no alexithymia, 51–60 indicating possible alexithymia, and ≥ 61 indicating alexithymia. Higher scores reflect more severe alexithymia. In the present study, the Cronbach’s α coefficient for this scale was 0.807.

#### 2.2.5 Difficulties in emotion regulation scale

The Chinese version of the 16-item Difficulties in Emotion Regulation Scale (DERS-16), revised by Wang and colleagues (27), was used to assess difficulties in emotion regulation. This scale comprises six dimensions and 16 items rated on a 5-point Likert scale, with higher scores indicating greater difficulties in emotion regulation. In the present study, the Cronbach’s α coefficient for this scale was 0.916.

### 2.3 Procedure and quality control

Two psychology graduate students served as research assistants. Paper-and-pencil questionnaires were administered in a classroom setting. The research assistants explained the purpose of the study, instructions for completing the questionnaire, and relevant precautions. Completed questionnaires were collected immediately after all items had been filled out. Questionnaires with patterned or careless responding were excluded.

### 2.4 Data analysis

Data were entered and organized using Microsoft Excel 2019. All statistical analyses were performed using IBM SPSS Statistics (Version 27.0). Descriptive statistics and Pearson correlation coefficients were computed to characterize the sample and examine associations among study variables.

To test the hypothesized chain mediation model, we used the PROCESS macro (Version 4.2) for SPSS developed by Hayes (28). The significance of all indirect effects was assessed using bias-corrected bootstrapping with 5,000 resamples (28). An indirect effect was considered statistically significant if its 95% bias-corrected confidence interval did not contain zero. This bootstrapping approach is recommended for mediation analysis because it does not assume normality of the sampling distribution of indirect effects and provides more accurate Type I error rates and greater statistical power than traditional methods such as the Sobel test (29).

To test the robustness of the findings and control for potential confounding variables, we included gender, academic year, family residence, family structure, and BMI as covariates in the mediation model. The selection of covariates was based on demographic comparisons showing that academic year, family residence, family structure, and BMI had significant effects on childhood emotional neglect and alexithymia, while extensive research has demonstrated that gender exerts a significant influence on emotional eating (30, 31). Therefore, these five demographic variables were incorporated into the serial mediation model in this study.

## 3 Results

### 3.1 Common method bias

As all data were collected through self-report measures, Harman’s single-factor test was used to assess potential common method bias. An unrotated exploratory factor analysis yielded 17 factors with eigenvalues greater than 1. The first factor explained 20.19% of the total variance, which is below the commonly used critical value of 40% (32), suggesting that common method bias is not a serious concern in this study. However, Harman’s single-factor test is only a relatively crude diagnostic method and cannot completely rule out the potential influence of common method bias; therefore, the relevant results should still be interpreted with caution.

### 3.2 Descriptive statistics and demographic differences

Independent-samples *t*-tests and one-way analysis of variance (ANOVA) were conducted to examine whether study variables differed across demographic characteristics. The results indicated that alexithymia scores differed significantly by academic year (*P* < 0.001); *post-hoc* comparisons revealed that first-year students reported higher alexithymia scores than second-year, third-year, and fourth-year students. Alexithymia scores also differed significantly by family residence (*P* < 0.001), with students from rural areas scoring higher than those from urban areas. Additionally, alexithymia scores differed significantly by BMI (P < 0.05), with obese individuals scoring higher than those who were underweight, within the normal range, and overweight. Childhood emotional neglect scores differed significantly by family structure (*P* < 0.001), specifically, students from divorced families reported higher scores than those from intact, restructured, and other family structures see **Table 1**

**Table 1:** Comparison of differences among variables on demographic characteristics.

Demographic variables	n		CEN score	EES score	TAS score	DERS score
Gender	Male	288	9.32 ± 4.15	63.48 ± 16.27	54.12 ± 10.04	41.83 ± 10.58
	Female	573	9.82 ± 4.16	61.85 ± 15.87	53.32 ± 9.72	42.06 ± 10.09
	T		-1.675	1.412	1.126	-0.037
	P		0.094	0.158	0.261	0.759
Academic year	Year 1	338	9.54 ± 3.89	63.01 ± 16.74	55.27 ± 10.17	42.89 ± 10.28
	Year 2	250	9.33 ± 4.03	61.48 ± 15.40	51.86 ± 9.31	40.97 ± 9.64
	Year 3	171	10.29 ± 4.64	63.18 ± 15.61	53.91 ± 9.12	41.94 ± 10.15
	Year 4	102	9.74 ± 4.41	61.25 ± 15.72	51.69 ± 10.12	41.98 ± 10.25
	F		1.961	0.748	7.360	1.765
	P		0.118	0.523	<0.001	0.152
	LSD				Year1>Year2, Year1>Year4, Year2<Year3	
Family residence	Rural areas	569	9.81 ± 4.12	62.26 ± 15.41	54.42 ± 9.55	42.43 ± 9.93
	Urban areas	292	9.33 ± 4.22	62.66 ± 17.15	51.97 ± 10.17	41.12 ± 10.80
	T		1.610	-0.348	3.490	1.769
	P		0.108	0.728	<0.001	0.077
Only-children	Yes	98	9.11 ± 4.14	61.35 ± 17.35	52.37 ± 9.78	42.05 ± 9.57
	No	763	9.71 ± 4.16	62.53 ± 15.84	53.74 ± 9.83	41.98 ± 10.34
	T		-1.361	-0.686	-1.305	0.068
	P		0.174	0.493	0.192	0.946
Demographic variables	n		CEN Score	EES Score	TAS Score	DERS Score
Family structure	Original family	740	9.47 ± 4.06	62.61 ± 15.94	53.52 ± 9.74	42.04 ± 10.19
	Divorced family	70	11.60 ± 4.82	60.49 ± 15.91	54.50 ± 9.31	42.30 ± 10.53
	Reconstituted family	39	9.92 ± 4.06	61.72 ± 16.43	53.28 ± 11.25	40.51 ± 10.53
	Others	12	8.75 ± 3.52	62.39 ± 16.01	53.50 ± 13.53	41.33 ± 12.37
	F		5.956	0.398	0.226	0.314
	P		<0.001	0.754	0.878	0.816
	LSD		Divorced>Original; Divorced>ReconstituteDivorced>Others			
BMI	Underweight	190	9.81 ± 4.18	60.63 ± 14.84	54.22 ± 9.55	42.49 ± 10.19
	Normal range	556	9.60 ± 4.12	62.73 ± 16.24	53.10 ± 9.79	41.75 ± 10.15
	Overweight	94	9.46 ± 4.43	63.61 ± 16.81	53.95 ± 10.25	41.44 ± 10.94
	Obesity	21	10.38 ± 4.03	63.90 ± 16.32	59.05 ± 9.87	45.95 ± 9.89
	F		0.395	1.098	2.938	1.392
	P		0.757	0.349	0.032	0.244
	LSD				Obesity>Underweight Obesity>Normal range Obesity>Overweight	

### 3.3 Correlation analysis

Correlation analyses revealed significant associations among alexithymia, difficulties in emotion regulation, and emotional eating (*P* < 0.01). However, the bivariate correlation between childhood emotional neglect and emotional eating was not significant (*r* = 0.011, *P* > 0.05). Contemporary mediation analysis has demonstrated that a significant indirect effect may exist even when the zero-order correlation is nonsignificant, particularly in the presence of suppression effects or inconsistent mediation (33). Accordingly, we proceeded with subsequent analyses as planned, with full acknowledgment of this preliminary nonsignificant relationship see **Table 2**.

**Table 2:** Descriptive statistics and correlation analysis of variables.

	M ± SD	CEN	EES	TAS	DERS
CEN	9.65 ± 4.16	1			
EES	62.39 ± 16.01	0.011	1		
TAS	53.59 ± 9.83	0.242^**^	0.160^**^	1	
DERS	41.98 ± 10.25	0.233^**^	0.169^**^	0.609^**^	1

** indicates p < 0.01

### 3.4 Chain mediation analysis

To assess the relationship between childhood emotional neglect and emotional eating, this study employed Model 6 of the PROCESS macro module for SPSS, which is designed for analyzing serial multiple mediation models. Childhood emotional neglect was included as the independent variable, emotional eating as the dependent variable, and alexithymia and emotion regulation difficulties as mediator variables in the serial mediation model. Gender, grade, hometown, family structure, and BMI were entered as covariates. The Bootstrap method was used, with 5,000 resamples drawn to evaluate the mediation effects, and a 95% confidence interval was maintained.

**Figure 1:**
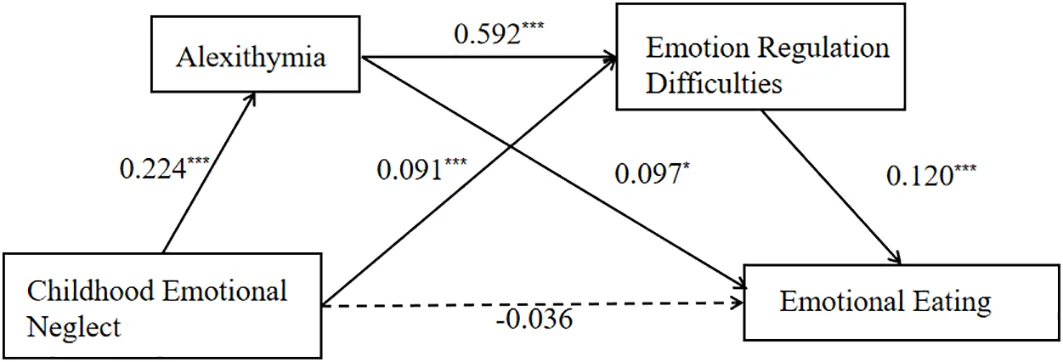
Results of path coefficient testing for the chain mediating effect of alexithymia and difficulties in emotion regulation between childhood emotional neglect and emotional eating.

The results showed that the total indirect effect of childhood emotional neglect on emotional eating was significant (effect = 0.1994, Boot SE = 0.0475, 95% CI [0.1118, 0.2985]), indicating that alexithymia and emotion regulation difficulties played a mediating role in this relationship. Specifically, all three indirect paths reached significance: (1) the specific indirect path via alexithymia alone was significant (X→M1→Y: effect = 0.0906, Boot SE = 0.0450, 95% CI [0.0034, 0.1832]), accounting for 45.44% of the total indirect effect; (2) the specific indirect path via emotion regulation difficulties alone was significant (X→M2→Y: effect = 0.0419, Boot SE = 0.0213, 95% CI [0.0060, 0.0895]), accounting for 21.01% of the total indirect effect; and (3) the serial mediation path of “childhood emotional neglect → alexithymia → emotion regulation difficulties → emotional eating” was also significant (X→M1→M2→Y: effect = 0.0668, Boot SE = 0.0296, 95% CI [0.0124, 0.1306]), accounting for 33.50% of the total indirect effect. In addition, the direct effect of childhood emotional neglect on emotional eating was not significant (effect = −0.1373, Boot SE = 0.1349, 95% CI [−0.4021, 0.1275]), and the total effect was also not significant (effect = 0.0621, Boot SE = 0.1321, 95% CI [−0.1972, 0.3214]). The results are presented in **Tables 3**, **4**, and **Figure 1**.

**Table 3:** Regression analysis of the relationship between childhood emotional neglect and emotional eating.

Predictor variable	TAS					DERS					EES				
	β	Std.β	t	LLCI	ULCI	β	Std.β	t	LLCI	ULCI	β	Std.β	t	LLCI	ULCI
Gender	-0.869	-0.042	-1.222	-2.264	0.526	0.577	0.027	0.944	-0.623	1.776	-1.038	-0.031	-0.872	-3.374	1.298
academic year	-1.079	-0.113	-3.441^***^	-1.695	-0.464	0.105	0.011	0.386	-0.428	0.637	0.021	0.001	0.041	-1.015	1.058
Family residence	-2.316	-0.112	-3.400^***^	-3.652	-0.979	0.366	0.017	0.622	-0.790	1.522	0.865	0.026	0.754	-1.386	3.115
Family structure	-0.082	-0.005	-0.149	-1.162	0.999	-0.579	-0.033	-1.224	-1.507	0.349	-0.482	-0.018	-0.524	-2.291	1.326
BMI	0.238	0.016	0.464	-0.768	1.244	-0.092	-0.006	-0.208	-0.956	0.773	1.093	0.044	1.275	-0.590	2.775
CEN	0.577	0.244	7.419^***^	0.424	0.729	0.224	0.091	3.245^***^	0.088	0.359	-0.137	-0.036	-1.018	-0.402	0.128
TAS						0.617	0.592	20.993^***^	0.559	0.675	0.157	0.097	2.231^*^	0.019	0.296
DERS											0.188	0.120	2.816^***^	0.057	0.319
R	0.293					0.617					0.200				
R2	0.086					0.381					0.040				
the F-statistic	13.398^***^					74.984^***^					4.444^***^				

^*^ P<0.05,^**^P<0.01,^***^P<0.001.

**Table 4:** Chain mediation of alexithymia and emotion regulation difficulties in the relationship between childhood emotional neglect and emotional eating.

Paths	Effect size	Boot SE	95%CI	Proportion of total indirect effect
Indirect effect	0.1994	0.0475	[0.1118,0.2985]	–
X→M1→Y	0.0906	0.0450	[0.0034,0.1832]	45.44%
X→M2→Y	0.0419	0.0213	[0.0060,0.0895]	21.01%
X→M1→M2→Y	0.0668	0.0296	[0.0124,0.1306]	33.50%
Direct effect	-0.1373	0.1349	[-0.4021,0.1275]	–
Total effect	0.0621	0.1321	[-0.1972,0.3214]	–

### 3.5 Multicollinearity test

To examine whether severe multicollinearity existed among the predictor variables in the model, a collinearity diagnosis was further conducted. The results showed that the tolerance values of all predictor variables ranged from 0.603 to 0.918, and the VIF values ranged from 1.090 to 1.659, all of which were below the commonly used thresholds. This indicates that there was no serious multicollinearity problem in the model see **Table 5**.

**Table 5:** Multicollinearity diagnostics for predictors included in the final model.

Predictor	Tolerance	VIF
Childhood emotional neglect	0.918	1.090
Alexithymia	0.603	1.659
Difficulties in emotion regulation	0.619	1.615

Tolerance values ranged from 0.603 to 0.918, and VIF values ranged from 1.090 to 1.659, indicating no evidence of serious multicollinearity.

### 3.6 Model robustness and alternative model

To further examine the robustness of the serial mediation sequence, this study constructed an alternative serial mediation model by reversing the order of alexithymia and emotion regulation difficulties. The results showed that the serial mediation effect of “CEN→TAS→DERS→EES” in the hypothesized model was significant (effect = 0.0668, Boot SE = 0.0296, 95% CI [0.0124, 0.1306]), whereas the serial mediation effect of “CEN→DERS→TAS→EES” in the alternative model was not significant (effect = 0.0503, Boot SE = 0.0263, 95% CI [−0.0003, 0.1037]). These findings indicate that, in the current data, the sequence in which alexithymia precedes emotion regulation difficulties received relatively stronger statistical support see **Table 6**.

**Table 6:** Robustness and alternative model analyses.

Model	Indirect pathway	Effect	BootSE	95% CI	Significant
Hypothesized sequential mediation model	CEN→TAS→DERS→EES	0.0668	0.0296	[0.0124,0.1306]	Yes
Alternative sequential mediation model	CEN→ DERS→TAS →EES	0.0503	0.0263	[-0.0003, 0.1037]	No

Both models controlled for gender, academic year, family residence, family structure, and BMI. Indirect effects were estimated using 5,000 bias-corrected bootstrap samples.

## 4 Discussion

### 4.1 Nonsignificant total and direct effects of childhood emotional neglect on emotional eating

The present study found that neither the total effect nor the direct effect of childhood emotional neglect on emotional eating was significant, whereas the total indirect effect was significant. This finding is inconsistent with some studies that have reported a significant direct association between childhood emotional neglect and emotional eating (34, 35), but aligns with other research emphasizing the role of mediating mechanisms (9, 15). These results suggest that the association between childhood emotional neglect and emotional eating may not be reflected in a direct relationship, but may instead be accounted for by intermediate psychological factors.

This result may be related to the characteristics of the sample selected in this study. First, the participants were medical students, who exhibited relatively low levels of childhood emotional neglect overall. The sample was predominantly female, and most participants’ BMI fell within the normal or underweight range, with low proportions of overweight and obesity, suggesting that this population may not represent a high-risk sample for weight-related eating problems. In addition, medical students typically possess higher levels of health knowledge and stronger behavioral norm awareness (36), which may have attenuated the direct correspondence between emotional neglect and emotional eating to some extent. The zero-order correlation between childhood emotional neglect and emotional eating in this study was only 0.011, also supporting this weak association pattern.

From the perspective of the mediation analysis results, this study provides greater support for an “indirect-only mediation” pattern (37), suggesting that childhood emotional neglect may be associated with emotional eating primarily through the proposed mediating variables rather than through a direct association. It should be noted that a nonsignificant total effect does not preclude the existence of mediation. Contemporary mediation analysis holds that the significance of the indirect effect is the primary criterion for establishing mediation. When the independent variable and dependent variable lack a significant direct association but are linked through significant indirect pathways, this pattern is consistent with an indirect-dominant association pattern (38). Thus, the present findings do not imply that childhood emotional neglect is unrelated to emotional eating; rather, they suggest that this association may be primarily accounted for by internal psychological factors. However, the non-significant total effect also suggests that the overall association between the two variables may be relatively weak in this sample, or may be subject to the combined influence of multiple mechanisms operating in different directions. Therefore, the interpretation of the indirect effects still requires cautious consideration in conjunction with theoretical background, sample characteristics, and potential omitted variables.

Furthermore, in this study, the direct effect of childhood emotional neglect on emotional eating was negative, whereas the total indirect effect was positive; the two effects were in opposite directions and numerically offset each other, which may be an important reason why the total effect failed to reach significance. This suggests the possible presence of inconsistent mediation or a suppression effect (28). One plausible explanation is that, in a predominantly female sample of medical students with normal or underweight BMI, some individuals who experienced higher levels of emotional neglect may not regulate their emotions through eating, but may instead be more inclined to adopt strategies such as emotional suppression, need avoidance, or weight control to cope with internal discomfort (37). These potential variables may help explain why the direct association between childhood emotional neglect and emotional eating appeared negative in this sample. However, since this direct effect did not reach statistical significance, and this study employed a cross-sectional design, the directional findings still need to be further verified in future research.

From a theoretical perspective, childhood emotional neglect, as an early adverse experience (39), may not show a direct association with specific behaviors. Instead, it may be associated with long-term differences in individuals’ emotional socialization processes, including patterns of emotion identification, expression, and regulation (40). In contrast, emotional eating is a behavior that is more closely associated with current situational factors. Beyond early experiences, it is also associated with momentary emotional states, dietary restraint, body image, and environmental cues (41–44). Therefore, the absence of a significant direct association between childhood emotional neglect and emotional eating is theoretically understandable. Unlike more overt forms of abuse, emotional neglect is characterized by the chronic absence of emotional responsiveness and support; accordingly, it may be more closely associated with emotion-processing difficulties than with a directly observable behavioral problem. It should be further noted that the mediation effects in this study primarily reflect an indirect association pattern consistent with theoretical expectations, rather than direct evidence of the temporal or causal ordering of the variables. Future research may consider employing longitudinal tracking to further examine why childhood emotional neglect may manifest in different behavioral outcomes across individuals, as well as the dynamic roles played by alexithymia and emotion regulation difficulties in this process.

### 4.2 The mediating role of alexithymia

The present study found that alexithymia showed a significant indirect effect in the association between childhood emotional neglect and emotional eating. This finding suggests that childhood emotional neglect may be associated with a greater tendency toward emotional eating through its association with difficulties in emotion identification, differentiation, and expression.

This result can be explained from the perspective of emotional socialization theory. The development of children’s emotional competence depends on caregivers’ sensitive responses to their emotional experiences, emotional mirroring, and verbal labeling (45). When children are chronically exposed to environments in which their emotional needs are unseen and unresponded to, their internal emotional experiences may be less likely to become integrated and represented, which may in turn be associated with elevated alexithymia (46). Previous research has also documented a stable association between emotional neglect and alexithymia, and between alexithymia and various maladaptive behaviors (46).

With respect to emotional eating, alexithymia may be linked to it through at least two possible processes. First, when individuals have difficulty clearly identifying and expressing negative emotions, they may be more likely to rely on external, immediate means of coping with discomfort; eating, given its accessibility and rewarding properties, may therefore become a common choice (17). Second, individuals with alexithymia may have difficulty distinguishing emotional arousal from interoceptive bodily signals, which may increase the likelihood of misinterpreting experiences of anxiety, emptiness, or tension as hunger and may be associated with greater emotional eating (47). Thus, alexithymia may be an important emotion-processing factor associated with the link between early emotional neglect and maladaptive eating behavior.

Notably, the independent mediating effect of alexithymia accounted for a substantial proportion of the total indirect effect in the present study, suggesting that difficulties in emotion identification and expression may play a particularly important role in the observed association. This finding implies that understanding the development of emotional eating requires attention not only to whether individuals can control their eating behavior, but also to whether they possess the capacity to accurately identify and articulate their emotional experiences.

This mediation result not only reflects the current cross-sectional association but can also be understood within a broader developmental framework. Early emotional neglect is often accompanied by a deficient family emotional environment. Existing research has shown that caregivers’ (especially mothers’) own alexithymia and cognitive inflexibility can limit their ability to interpret and respond to children’s emotional signals. Children growing up in such family environments may have greater difficulty acquiring flexible emotion regulation strategies and may also be more likely to rely on food for emotional soothing; these patterns have been associated with higher levels of emotional eating and obesity in childhood (48, 49). Therefore, the association pattern observed in this study may reflect not only individual differences in emotional processing but also a broader development context in which the family emotional environment is related to later eating-related behaviors across developmental stages.

### 4.3 The mediating role of difficulties in emotion regulation

The present study further found that difficulties in emotion regulation showed a significant indirect effect in the association between childhood emotional neglect and emotional eating. This result is consistent with existing research (34), which indicates that early adverse experiences may be associated with poorer emotion regulation functioning, and that difficulties in emotion regulation may be closely related to emotional eating.

Difficulties in emotion regulation typically encompass multiple dimensions, including insufficient emotional awareness, difficulty accepting emotions, lack of regulatory strategies, and impaired impulse control (20). For individuals with weaker emotion regulation capacities, negative emotions may be associated with greater difficulty in employing adaptive strategies such as delay of gratification, cognitive reappraisal, or problem solving, and with a greater tendency to select short-term strategies that rapidly reduce discomfort (50). Eating, particularly of highly palatable foods, has been described as serving both negative and positive reinforcing functions, insofar as it may alleviate negative affect and provide immediate pleasure (51); accordingly, it may be more commonly used as a substitute coping strategy by individuals with difficulties in emotion regulation.

The present findings suggest that the association between childhood emotional neglect and emotional eating may involve not only difficulties in emotion identification, but also difficulties in emotion management and strategy use. This suggests that emotional eating may be related not merely to whether emotions are present, but more specifically to how individuals process their emotions.

### 4.4 The chain mediating effect of alexithymia and difficulties in emotion regulation

One of the most important findings of the present study was the observation of a significant sequential indirect association involving alexithymia and difficulties in emotion regulation in the association between childhood emotional neglect and emotional eating. This result is consistent with a sequential pattern in which childhood emotional neglect is associated with elevated alexithymia, alexithymia is associated with greater difficulties in emotion regulation, and these difficulties are further associated with higher levels of emotional eating. This chain pathway is consistent with a potential sequential pattern linking early emotional deprivation with current behavioral problems.

Theoretically, although alexithymia and difficulties in emotion regulation are closely related, they correspond to different levels of emotional functioning. The former primarily involves the identification, differentiation, and expression of emotions, and may be understood as relating more closely to emotion processing (16), whereas the latter reflects difficulties in the acceptance, control, and strategic deployment of emotions following activation (20), and may be more closely related to behavioral responding. Based on this functional distinction, effective emotion regulation is generally presumed to rest upon relatively clear emotional awareness and representation. If individuals struggle to identify what emotions they are experiencing, their selection and implementation of regulatory strategies may also become more difficult. Thus, the chain pathway identified in the present study is theoretically plausible.

Furthermore, in terms of relative effect sizes, the independent mediating pathway through alexithymia accounted for the largest proportion, followed by the chain pathway, with the independent mediating pathway through difficulties in emotion regulation being relatively smaller. This pattern suggests that difficulties in emotion identification and expression may correspond to an earlier theoretical stage in the observed association and may be closely related to difficulties in emotion regulation. In other words, the association between childhood emotional neglect and emotional eating may not be directly reflected in eating behavior itself, but may instead be linked through cumulative difficulties in emotional functioning.

However, it must be emphasized that because the present study employed a cross-sectional design, the chain pathway described above should be interpreted as a theoretically consistent pattern of associations rather than an established causal sequence. Bidirectional relationships may exist between alexithymia and difficulties in emotion regulation, and their structural interrelations may vary across different samples. Future research should employ longitudinal designs, cross-lagged panel models, or competitive model comparisons to further examine the temporal ordering and stability of this chain pathway.

Previous research has largely treated alexithymia and emotion regulation difficulties as parallel risk factors, with limited attention to their sequential relationship in the emotional processing chain (52, 53). This study, to some extent, extends this parallel perspective by examining a theoretically derived sequential association pattern, thereby contributing to a more differentiated understanding of emotion-related factors associated with emotional eating. However, given the cross-sectional design employed in this study, the above serial pathway should be more appropriately understood as an association pattern consistent with theory, rather than a confirmed causal sequence. There may be a bidirectional relationship between alexithymia and emotion regulation difficulties, and the two may exhibit different structural relationships across different samples. Future research should adopt longitudinal designs, cross-lagged panel models, or competitive model comparisons to further examine the temporal ordering and stability of this serial pathway.

### 4.5 Clinical and practical implications

The present findings have several implications for interventions targeting emotional eating. First, for individuals with a history of childhood emotional neglect, interventions focused solely on dietary control may be insufficient; attention to difficulties in emotion identification and emotion regulation may also be warranted. Second, alexithymia and difficulties in emotion regulation represent promising targets for intervention: the former may be ameliorated through training in emotional awareness, emotional labeling, and interoceptive discrimination; the latter may be addressed through training in emotional acceptance, cognitive reappraisal, impulse control, and alternative coping strategies. Finally, given that emotional neglect itself involves the absence of early emotional responsiveness, interventions may benefit from emphasizing supportive relationships and the reconstruction of a sense of safety. These intervention strategies may inform tiered approaches to treating emotional eating, although their effectiveness awaits further empirical validation.

### 4.6 Limitations and future directions

This study has the following limitations, which await further refinement in future research.

First, this study employed a cross-sectional design, with all variables measured at a single time point; therefore, it is difficult to make strict judgments regarding the causal relationships, temporal ordering, and directional effects among the variables. In particular, the serial mediation pathway examined in this study primarily reflects an association structure consistent with theoretical assumptions, rather than an actual causal transmission chain in the developmental process. Future research should employ longitudinal tracking, cross-lagged panel models, or experimental designs to further verify the dynamic relationships among the variables.

Second, all data in this study were derived from self-report scales, which may be subject to common method bias, social desirability, and recall bias. Specifically, retrospective reports of childhood emotional neglect may be influenced by current psychological states. Although this study conducted a preliminary assessment of common method bias using Harman’s single-factor test, and the results indicated no serious bias, this method itself is relatively crude and cannot completely rule out the influence of common method bias inherent in self-report data. Future research may incorporate multi-timepoint measurements, multiple informants, behavioral tasks, physiological indicators, or the introduction of method factors in statistical analyses to further enhance the robustness of the research conclusions.

Third, alexithymia and emotion regulation difficulties exhibit some conceptual and measurement overlap. Although the two are theoretically distinguishable, they may share some variance in empirical analyses, thereby affecting the identification of their unique effects. In addition, this study did not include important variables such as anxiety, depression, perceived stress, dietary restraint, and body image, which may simultaneously influence emotional eating; therefore, the current model may still contain potential omitted variables or competing pathways. Future research may employ structural equation modeling, latent variable analysis, or the inclusion of additional covariates, competitive mediators, and moderators to more finely examine the unique effects among the variables.

Fourth, this study used the total score of emotional eating as the dependent variable, primarily investigating the psychological mechanisms of its overall level. However, emotional eating under different emotional states may involve different formation mechanisms. Future research may conduct more systematic comparisons of the formation mechanisms of emotional eating across different emotional types based on dimensional differences.

Fifth, the sample in this study consisted primarily of non-clinical medical students from the Guizhou region, with a high proportion of females and BMI predominantly distributed within the normal or underweight range, with low proportions of overweight and obese individuals. Therefore, the results of this study are more applicable to explaining the psychological mechanisms of emotional eating in general college student populations, especially relatively low-risk samples, and caution should be exercised when generalizing to overweight/obese populations, metabolically high-risk groups, or clinical samples. Meanwhile, the regional origin, professional background, gender composition, and weight distribution of this study’s sample may also have influenced, to some extent, the strength of the association between childhood emotional neglect and emotional eating, as well as the manifestation of the mediation pathways. Future research is warranted to further test the present research model in samples from different regions, at different age stages, with different professional backgrounds, more balanced gender structures, and among overweight/obese or clinical populations, in order to enhance the external validity and generalizability of the research conclusions.

## 5 Conclusions

The present study found that although childhood emotional neglect was not directly associated with emotional eating, it showed significant indirect associations through alexithymia and difficulties in emotion regulation, which together were consistent with a significant sequential indirect association patten. These findings suggest that the association between childhood emotional neglect and emotional eating may be reflected in a theoretically derived sequential pattern involving difficulties in emotion identification and emotion regulation. This contributes to understanding factors associated with emotional eating from the perspective of emotion processing and regulation, and offers preliminary implications for future intervention research targeting emotion identification and emotion regulation.

## Data Availability

The raw data supporting the conclusions of this article will be made available by the authors, without undue reservation.

## Appendix

**Table 1:** Chain mediation of alexithymia and emotion regulation difficulties in the relationship between childhood emotional neglect and depression-related emotional eating.

Paths	Effect size	Boot SE	95%CI	t	p
Indirect effect	0.1007	0.0228	[0.0579,0.1488]	–	–
X→M1→Y1	0.0413	0.0214	[-0.0014,0.0831]	–	–
X→M2→Y1	0.0229	0.0105	[0.0050,0.0462]	–	–
X→M1→M2→Y1	0.0365	0.0146	[0.0106,0.0680]	–	–
Direct effect	-0.0324	0.0635	[-0.1571,0.0923]	-0.5099	0.6103
Total effect	0.0683	0.0624	[-0.0557,0.1894]	1.0945	0.2740

**Table 2:** Chain mediation of alexithymia and emotion regulation difficulties in the relationship between childhood emotional neglect and anxiety-related emotional eating.

Paths	Effect size	Boot SE	95%CI	t	p
Indirect effect	0.0361	0.0101	[0.0174,0.0567]	–	–
X→M1→Y	0.0235	0.0107	[0.0033,0.0445]	–	–
X→M2→Y	0.0049	0.0042	[-0.0028,0.0137]	–	–
X→M1→M2→Y	0.0078	0.0064	[-0.0043,0.0212]	–	–
Direct effect	-0.0042	0.0304	[-0.0638,0.0555]	-0.1371	0.8910
Total effect	0.0320	0.0296	[-0.0261,0.0900]	1.0811	0.2800

**Table 3:** Chain mediation of alexithymia and emotion regulation difficulties in the relationship between childhood emotional neglect and hostility-related emotional eating.

Paths	Effect size	Boot SE	95%CI	t	p
Indirect effect	0.0478	0.0128	[0.0237,0.0738]	–	–
X→M1→Y	0.0353	0.0126	[0.0114,0.0613]	–	–
X→M2→Y	0.0048	0.0048	[-0.0044,0.0153]	–	–
X→M1→M2→Y	0.0077	0.0075	[-0.0061,0.0237]	–	–
Direct effect	0.0163	0.0376	[-0.0575,0.0902]	0.4340	0.6644
Total effect	0.0642	0.0367	[-0.0079,0.1362]	1.7480	0.0808

**Table 4:** Chain mediation of alexithymia and emotion regulation difficulties in the relationship between childhood emotional neglect and positive-related emotional eating.

Paths	Effect size	Boot SE	95%CI	t	p
Indirect effect	0.0147	0.0105	[-0.0049,0.0367]	–	–
X→M1→Y	-0.0095	0.0108	[-0.0311,0.1117]	–	–
X→M2→Y	0.0093	0.0053	[0.0010,0.0212]	–	–
X→M1→M2→Y	0.0419	0.0070	[0.0023,0.0298]	–	–
Direct effect	-0.1171	0.0347	[-0.1851,-0.0490]	-3.3747	0.0008
Total effect	-0.1023	0.0335	[-0.1681,-0.0366]	-3.0552	0.0023
